# Drunkenness Statistics

**Published:** 1920-08-07

**Authors:** 


**THE PUBLIC WELL-BEING**—(*continued*)

### Drunkenness Statistics.

### THE DIFFICULTY OF DRAWING DEDUCTIONS.

The licensing statistics issued in an official blue book show a large increase of drunkenness in 1919 as compared with 1918. The total number of convictions in England and Wales was nearly double that in 1918, the figures being 57,948, as against 29,075 last year. The report is very cautious in its comments. It says that more or less superficial manipulation of the figures and facts on paper has all the dangers and sterility of mere speculation. Out of the many and various contributory circumstances it is almost impossible and extremely unsafe to isolate this one or that one, or this or that group, and call it the cause of the particular movement which may be under consideration. Some of the circumstances or causes—which operated at different times, in different ways, and in different degrees in different districts—may be stated as follows:—There were more men at home, and fewer of them in khaki, more policemen (and those less overworked) available for street duty, more hours for drinking, more (and stronger) liquor, more light in the streets, more money, more leisure, less self-control, less appreciation of the fact that drunkenness "matters" now the war is over, less readiness to realise that the progress towards general sobriety won during the war ought to be carried on in peace-time, and lack of adequate equipment for driving that point home.

Whatever the causes, the actual figures have been taken as a most unfortunate decline in national sobriety, as indeed they are. They seem to refute the declarations of labour leaders that more wages and shorter hours are demanded so that men may have leisure to enjoy the higher interests of life. A too hasty generalisation might lead to the assumption that more wages and shorter hours had meant more money to spend on beer and longer opportunities of drinking it. But this would not be a safe assumption. In the first place it seems certainly a reaction—passing or permanent—which comes with the removal of wartime restrictions of various kinds. In 1918—as a writer in *The Times* points out—every available man was mobilised, and the country was fighting for its life. In 1919, victory had been won, and a hundred and one forces came into play having direct bearing on drunkenness returns. A different tale is told if the comparative figures are extended backwards to before the war. The figures are these:—

| | | | |
|---|---|---|---|
| 1913 | 188,877 | 1917 | 46,410 |
| 1914 | 183,828 | 1918 | 29,075 |
| 1915 | 135,811 | 1919 | 57,948 |
| 1916 | 84,191 | | |

### Optimistic View.

We also find some support for a not too pessimistic outlook in two recent utterances which, though they do not directly concern the subject of drink, have direct relation to the cognate question of self-respect. It is always being declared that it is not possible to expect a decent race until indecent housing conditions have been swept away. That is true, yet there is another side of the matter to which those who know have drawn attention. Dr. Robertson, Medical Officer of Health for Birmingham, said recently that he thought it was perfectly wonderful that we get so many decent self-respecting men and women coming out of the slums. He is certain that under good conditions very many more would be forthcoming, and his experience among slum-dwellers leads him to hope for a vast improvement in the near future. Mrs. George Cadbury has expressed similar astonishment at the way many women rise above their surroundings, and she has spoken from her personal experience of the incalculable influence exercised by favourable environment. She has watched the process of transformation in families who have moved out of congested areas into better places. There will always be those who will be content to go on "pigging it," whatever their means and opportunities, but there are sufficient numbers of the opposite kind to make the effort at better all-round social conditions worth while.

Too much stress can be laid on the relation of drink to bad material environment, and for that reason it seems doubtful whether the advocacy of brighter and better drinking-houses—excellent in itself—is a very substantial contribution to the question of drunkenness. Why should anyone necessarily prefer to become intoxicated in a dismal public-house rather than in a bright one? To drink at all must certainly be more attractive in more attractive places, and the brightening process might

**THE PUBLIC WELL-BEING**—(*continued*).

just as well increase drinking as decrease it. It would not be rash to hazard a guess that there are records of drunkenness even in most gorgeous surroundings. We will not enter into the interminable discussions of phrases about enforcing sobriety by law, and all the rest, but there is one practical step which might with advantage be taken, and that is to stiffen the inadequate penalties now imposed upon those who make themselves disgusting public nuisances, and upon those whose conduct enables this to be done.

